# In-Vitro Study of Indium (III) Sulfate-Containing Medium on the Viability and Adhesion Behaviors of Human Dermal Fibroblast on Engineered Surfaces

**DOI:** 10.3390/ma16103814

**Published:** 2023-05-18

**Authors:** Ali Eskandari, Evelyn K. F. Yim, D. Moira Glerum, Ting Y. Tsui

**Affiliations:** 1 Department of Chemical Engineering, University of Waterloo, Waterloo, ON N2L 3G1, Canada; ali.eskandari@uwaterloo.ca (A.E.); eyim@uwaterloo.ca (E.K.F.Y.); 2 Waterloo Institute for Nanotechnology, University of Waterloo, Waterloo, ON N2L 3G1, Canada; 3 Department of Biology, University of Waterloo, Waterloo, ON N2L 3G1, Canada

**Keywords:** human dermal fibroblast cell, adhesion, indium sulfate, tantalum, biomaterial

## Abstract

Tissues and organs consist of cells organized in specified patterns that support their function, as exemplified by tissues such as skin, muscle, and cornea. It is, therefore, important to understand how external cues, such as engineered surfaces or chemical contaminants, can influence the organization and morphology of cells. In this work, we studied the impact of indium sulfate on human dermal fibroblast (GM5565) viability, production of reactive oxygen species (ROS), morphology, and alignment behavior on tantalum/silicon oxide parallel line/trench surface structures. The viability of cells was measured using the alamarBlue™ Cell Viability Reagent probe, while the ROS levels in cells were quantified using cell-permeant 2′,7′-dichlorodihydrofluorescein diacetate. Cell morphology and orientation on the engineered surfaces were characterized using fluorescence confocal and scanning electron microscopy. When cells were cultured in media containing indium (III) sulfate, the average cell viability decreased by as much as ~32% and the concentration of cellular ROS increased. Cell geometry became more circular and compact in the presence of indium sulfate. Even though actin microfilaments continue to preferentially adhere to tantalum-coated trenches in the presence of indium sulfate, the cells are less able to orient along the line axes of the chips. Interestingly, the indium sulfate-induced changes in cell alignment behavior are pattern dependent—a larger proportion of adherent cells on structures with line/trench widths in the range of 1 μm and 10 μm lose the ability to orient themselves, compared to those grown on structures with line widths smaller than 0.5 μm. Our results show that indium sulfate impacts the response of human fibroblasts to the surface structure to which they adhere and underscores the importance of evaluating cell behaviors on textured surfaces, especially in the presence of potential chemical contaminants.

## 1. Introduction

The adhesion and alignment of cells on biological surfaces, such as the extracellular matrix (ECM), are important for differentiation, signaling, cell growth, and migration. Some tissues are required to assume specific geometric patterns to function well [1], as seen with myoblasts, which become aligned into myotubes to form the muscular system [2]. Highly organized cells can also be found in the lens of the vertebrate eye [3]. In addition, the morphology, orientation, and function of skin cells can be modified by microenvironment and external cues, such as interstitial flow [4], tissue-scale tensional homeostasis [5], external electric field [6], mechanical strain [7], and even mobile phone signals [8]. Therefore, it is important to understand the impact of potential environmental contaminants, such as indium, on the morphology and function of cells when they are organized in different patterns. Models developed based on results collected from cells incubated on flat petri dish surfaces with randomly oriented morphology may not represent the real impact of indium (contaminants) within the cells of the organs and tissues of our body. 

Since the 2000s, there has been a proliferation of indium-containing consumer products, such as thin-film solar cells [9,10], organic light-emitting devices [11], flat panel displays [12], and advanced integrated circuits [13]. It is estimated that ~70% of the indium consumed is used to produce indium-tin-oxide (ITO) thin film for flat panel displays [12]. While the use of indium in consumer products has increased, the impacts of indium compounds on biological entities have not been well studied until recently [14,15,16]. Homma et al. [15] and Chonan et al. [16] suggested continuous inhalation of ITO particles may lead to interstitial pneumonia and other indium lung diseases. In an animal study performed by Takagi et al. [17], they observed an increased number of indium (III) chloride (InCl_3_)-induced micronuclei in BALB/c mice receiving 2.5 and 5 mg/kg body weight of InCl_3_, which is a possible indication of genotoxicity, while Ungváry et al. [18] found that a dose of 400 mg/kg InCl_3_ can be embryotoxic and teratogenic in rats.

At the cellular level, Tabei et al. [19] observed increased intracellular reactive oxygen species (ROS) and the inflammatory marker interleukin-8 after human lung epithelial (A549) cells were incubated with indium in ITO. In addition, indium oxide nanoparticles have been shown to cause cytotoxicity and apoptosis in human lung epithelial (A549) cells through mitochondrial membrane potential loss and the generation of ROS [20]. Indium (III) chloride-induced (InCl_3_) cytotoxicity, apoptosis, and genotoxicity on RAW264.7 mouse macrophages were studied by Tsai et al. [21], who found that InCl_3_ concentrations greater than 1 μM resulted in increased levels of apoptosis. Furthermore, micronuclei were found in cells treated in media containing more than 5 μM InCl_3_, a characteristic of InCl_3_-induced genotoxicity. Lee et al. [22] observed cytotoxicity in human oral keratinocytes when cells were exposed to high dosages of InCl_3_ or indium sulfate (In_2_(SO_4_)_3_). They showed that both indium compounds can decrease cell viability in media containing indium concentrations higher than 1.6 mM. At a concentration of 0.8 mM, indium can induce terminal differentiation in the keratinocytes. While these studies shed light on the toxicity of indium-containing compounds for mammalian cells, most studies have used InCl_3_ as the source of indium rather than In_2_(SO_4_)_3_; the impact of In_2_(SO_4_)_3_ on human dermal fibroblast remains unclear. Furthermore, most prior studies have shown the impact of indium on adherent cells on flat un-patterned surfaces, so there is little understanding of how oriented cells grown on patterned substrates behave when exposed to In_2_(SO_4_)_3_.

Although cells may orient or align within the self-produced ECM in tissues, the morphology of cells can also be manipulated by using artificial external cues, such as engineered nano-/micro-topographic surface features or surfaces constructed with dissimilar materials. These engineered structures have been useful in further understanding cell adhesion, differentiation, migration, and growth on surfaces. It has been demonstrated that adherent cells preferentially orient parallel to line/trench topographic structures constructed with polymer [23,24,25], tantalum [26], or tantalum/silicon oxide composites [27]. For example, Moussa et al. [28] used engineered tungsten/silicon oxide parallel line structures to investigate the effect of Antimycin A on adherent human fibroblast (GM5565) morphology. Antimycin A is a bio-toxin that can induce ROS and superoxide production [29] and create oxidative stress in cells. The mitochondrial morphology of GM5565 cells changes from tubular network to puncta when treated with Antimycin A—a possible indication of cellular oxidative stress [28]. The results show that, at a concentration of 5 μM, Antimycin A can induce degradation of the alignment of cells on parallel line structures; thus, cell alignment on parallel line structures can also serve as a potential indicator of cellular responses to cytotoxic compounds. While indium compounds, such as ITO [19], indium oxide [20], and InCl_3_ [21], can also induce ROS production in cells, the degree of In_2_(SO_4_)_3_-induced ROS production and the impacts on human fibroblast alignments on engineered surfaces is not well characterized.

The objective of this work was to develop an understanding of how In_2_(SO_4_)_3_ influences human dermal fibroblast morphology and adhesion behavior on flat Petri dishes and engineered surfaces with tantalum/silicon oxide patterned in parallel line/trench of equal widths. The engineered surface is used to induce cell alignment parallel to the line axes, and our results provide new insights into how In_2_(SO_4_)_3_ affects cells’ ability to orient and align on different surface topographies. Our work likely provides a more realistic representation of the impact of In_2_(SO_4_)_3_ within the human body. We suggest that the changes in cell alignment behavior on these engineered surfaces may represent a new way to assess the impacts of environmental contaminants on mammalian cell populations. The human dermal fibroblast cell line (GM5565) used in this study is untransformed and thus an appropriate material to better understand the chemical effect(s) of indium compounds on skin cells. While Eskandari et al. [30] and others have investigated the impacts of InCl_3_ on cell morphology and other cell behaviors, it is unclear if the same effects will be induced by In_2_(SO_4_)_3_. Understanding the impacts of In_2_(SO_4_)_3_ is explicitly important because, during the recycling of flat panel displays, indium can be recovered from ITO through a concentrated sulfuric acid leaching process [12]. Herein, the cellular response to media containing five different In_2_(SO_4_)_3_ concentrations was studied, with the concentrations covering the range of indium dosages (0 mM to 3.2 mM) that are known to induce cytotoxicity and apoptosis [22]. One of the unique features of the patterned tantalum-silicon oxide composite surface structures created for this work is their ability to manipulate cell morphology using both mechanotransduction response from cells and their selective adhesion characteristics on dissimilar material. Tantalum and its alloy exhibit excellent bio-compatibility with high mechanical strengths and are widely used as medical implants [31,32,33,34]. The morphology of cells and mitochondria within cells incubated in media containing In_2_(SO_4_)_3_ were inspected using fluorescence confocal microscopy, and the impact on cell viability, intracellular ROS production and cell alignment performance on engineered structures were measured. Our results indicate decreased cellular viability and increased ROS production when cells are incubated in a medium containing In_2_(SO_4_)_3_. The interplay between the line/trench width of the engineered structures and the concentration of In_2_(SO_4_)_3_ on adherent cell alignments was also analyzed, with our data showing the impact of In_2_(SO_4_)_3_ on cell alignment depending on the design of the parallel line structures. Specifically, the same indium dosage leads to decreased cell alignment with increasing line/trench width. Our results underscore the importance of investigating cell behaviors on engineered structures, in addition to the traditional studies using flat petri dish surfaces.

## 2. Materials and Methods

### 2.1. Substrates

**Figure 1:**
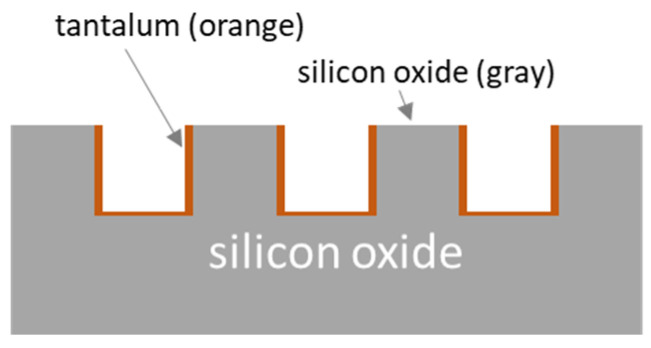
A schematic drawing of the surface structure fabricated in this work. The trench sidewall and bottom surfaces are coated with tantalum film, while the silicon oxide is exposed on the line top surface.

The influence of indium (III) sulfate (In_2_(SO_4_)_3_) on cell adhesion and alignment behavior was investigated on (i) 35 mm tissue culture dishes (Biolite 35 mm, Thermo Scientific, Mississauga, ON, Canada) and (ii) parallel line surface comb structures with equal line and trench widths of 0.18 μm, 0.25 μm, 0.5 μm, 1 μm, 2 μm, and 10 μm. A schematic cross-section drawing of the patterned comb structure is displayed in Figure 1. It shows the top surface of lines composed of silicon oxide, while the bottom and sidewall surfaces are coated with a thin layer of tantalum thin film. The comb structure fabrication processes have been described elsewhere. Each patterned area is rectangular, with a length of ~1.2 mm and a width of ~1.5 mm. 

### 2.2. Cell Culture and Plating on Patterned Substrates

Human dermal fibroblasts (GM5565; ATCC/Coriell Institute, Camden, NJ, USA) were cultured in T-50 flasks with 10 mL of media at 37 °C and 5% CO_2_ in Minimum Essential Medium Alpha (Gibco/Thermo Fisher Scientific, Waltham, MA, USA) supplemented with 10% (*v*/*v*) fetal bovine serum (FBS). No antibiotics were added to the media, given the demonstrated deleterious effects of aminoglycosides, such as streptomycin, on mitochondrial function [35].

The indium concentration in the baseline medium is less than 0.5 μM as quantified by inductively coupled plasma mass spectrometry techniques. Baseline medium was supplemented with anhydrous In_2_(SO_4_)_3_ (≥98.0%, Millipore Sigma, Burlington, MA, USA) to produce five different target indium concentrations of 0 mM, 0.4 mM, 0.8 mM, 1.6 mM, and 3.2 mM. Prior to cell seeding, patterned substrates were sterilized by submerging them in 2 mL of 70% ethanol for 1 min. They were then air-dried and rinsed with 2 mL of sterile phosphate-buffered saline (PBS) two times to remove any residue. Cell seeding was carried out by adding media containing 1–9 × 10^4^ cells/mL to substrates. Specimens were incubated in 6 well tissue culture plates (VWR^®^ Multiwell Cell Culture Plates (Radnor, PA, USA), 10062-892) for 24–48 h at 37 °C and 5% CO_2_. 

### 2.3. Cell Viability

The viability of cells in media containing the different indium concentrations was characterized by using alamarBlue™ Cell Viability Reagent (DAL1025, Thermo Fisher Scientific, Mississauga, ON, Canada); supplier recommended sample preparation protocol was followed. Briefly, ~2 × 10^5^ cells/mL medium (~6 × 10^4^ cells/cm^2^ areal coverage) were deposited into transparent Fisherbrand™ 96-Well flat bottom microplate (FB012931, Fisher Scientific, Ottawa, ON, Canada) and indium sulfate was then added to the wells to achieve the targeted indium concentrations. After incubation for 48 h at 37 °C and 5% CO_2_, media was gently aspirated from each well and replaced with 100 μL of diluted Alamarblue solution (10% *v/v* Alamarblue to indium-free baseline media). The 48 h incubation time was selected to align with similar cytotoxicity works reported in the literature [21,22,26,30]. After an additional 4 h of incubation (37 °C and 5% CO_2_), the absorbance in each well was measured at wavelengths of 570 nm and 600 nm, using a microplate reader (Biotek Synergy 4, Mississauga, ON, Canada.); data were analyzed according to Goegan et al. [36]. Experiments were carried out in triplicate using three biological replicates. 

### 2.4. Characterization of Cellular Reactive Oxygen Species (ROS)

The In_2_(SO_4_)_3_-induced cellular reactive oxygen species (ROS) levels were characterized by using the DCFDA/H2DCFDA-Cellular ROS Assay Kit (ab113851, Abcam Inc., Cambridge, MA, USA), according to the manufacturer-recommended protocol. Indium-free baseline media with ~1 × 10^5^ cells/mL was dispensed into a Nunc™ MicroWell™ 96-Well, Nunclon Delta-Treated, Flat-Bottom Microplate (Thermal Fisher Scientific, Mississauga, ON, Canada). Appropriate amounts of In_2_(SO_4_)_3_ supplement were added to each well and incubated for 24 h (37 °C, 5% CO_2_). Following incubation, media was aspirated, and samples were rinsed twice with PBS before introducing the diluted DCFDA (40 mM) into the wells. After 30 min of incubation, the wells were rinsed with 1× buffer solution. The fluorescence intensity of each well was measured using a microplate reader (Biotek Synergy H1, Mississauga, ON, Canada) and an excitation wavelength of 485 nm; emission intensity at 515 nm to 545 nm was measured and analyzed. Three different batches of cells were tested at each indium sulfate concentration. 

### 2.5. Fixation and Staining

The fixation and staining processes have been discussed in previous work [30]. After incubating on patterned substrates for 24–48 h, the spent media were aspirated, and the adherent cells were rinsed with PBS. The 24–48 h incubation time frame was selected to align with prior cytotoxicity studies [21,22,26,30]. Mitochondria in live cells were labelled by submerging the cell-coated substrates in 2 mL of 100 nM MitotrackerTM Red (Thermo Fisher Scientific, Mississauga, ON, Canada) at 37 °C for 20 min. Specimens were then rinsed with PBS and fixed with 4% paraformaldehyde for 15 min. Prior to further staining, cells were permeabilized for 5 min in 2 mL of 0.1% Triton-X (Sigma-Aldrich, St. Louis, MO, USA), followed by a PBS rinse. Phalloidin-iFluor 647 Reagent (ab176759, Abcam Inc, Cambridge, MA, USA) and 4′,6-diamidino-2-phenylindole (DAPI) (Life Technologies, Burlington, ON, Canada) were used to label F-actin microfilaments and DNA molecules, respectively. Specimens were rinsed multiple times with PBS after the application of each stain. Fluorescence confocal microscopy was performed using a Leica TCS SP5 confocal microscope (Leica, Wetzlar, Germany) located at the University of Guelph (Guelph, ON, Canada).

### 2.6. Scanning Electron Microscopy

Prior to inspections with scanning electron microscopy (SEM), adherent cells were fixed with paraformaldehyde as described in Section 2.5 and dried in sequential increasing concentrations of ethanol (50%, 75%, 95%, and 100%) for more than 10 min each. Cells were imaged with a high-resolution field emission scanning electron microscope (Zeiss 1550, Carl Zeiss AG, Oberkochen, Germany) with electron gun voltage operating at 7 kV. No electric conductive layer was coated on these samples to avoid artifacts.

## 3. Results and Discussion

### 3.1. Influence of Indium Sulfate on Adherent Cell Morphology 

**Figure 2:**
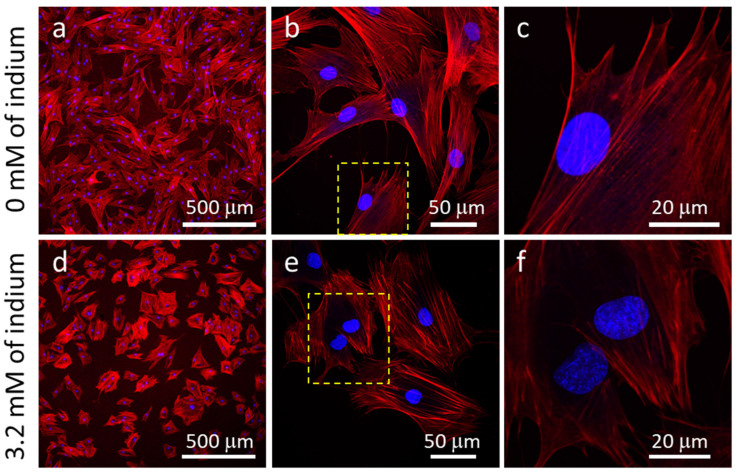
Confocal micrographs of adherent cells on flat surfaces cultured in (**a**–**c**) indium-free media and (**d**–**f**) media containing 3.2 mM of indium from indium sulfate precursor. The DNA molecules were labelled DAPI (blue), and actin microfilaments were stained with phalloidin (red).

A change in cell morphology is often one of the first indicators that cells have been exposed to a toxin; hence, it is useful to compare the morphology of cells when they are incubated in media with and without indium sulfate (In_2_(SO_4_)_3_). Typical fluorescence confocal micrographs of human dermal cells (GM5565) on flat culture dish surfaces incubated in medium without In_2_(SO_4_)_3_ and medium containing 3.2 mM of indium are shown in Figure 2a–f, respectively. The F-actin microfilaments appear red (phalloidin conjugate), while the DNA is stained blue (DAPI). The images show cells are randomly oriented after incubation in either medium, but there are visibly fewer adherent cells on the specimen treated with In_2_(SO_4_)_3_, suggesting a decrease in cell viability when cells are exposed to In_2_(SO_4_)_3_. The micrographs also reveal a change in cell morphology in the presence of indium, with the cells becoming more circular in shape and the actin fibres appearing more segmented. This is consistent with the change in GM5565 cell morphology observed by Eskandari et al. [30], where cells become more compact and circular when exposed to media containing 3.2 mM of InCl_3_. In contrast to a previous study by Tsai et al. [21], for InCl_3_-treated RAW264.7 mouse macrophages, there were no micronuclei observed in GM5565 cells after exposure to In_2_(SO_4_)_3_. This may suggest a different response to indium compounds in human and mouse cells or a difference in response between a somatic cell line (GM5565), which is untransformed, and a cancer cell line (RAW264.7), which is by definition transformed/immortalized.

The influence of In_2_(SO_4_)_3_ on the cell morphology was also quantified by characterizing the circularity index of cells. This index corresponds to the ratio between the cell projected area and the square of the cell perimeter length as defined by Equation (1). The circularity index has a theoretical value between 0 and 1, with a perfectly circular cell having an index value of one. Results show cells incubated in baseline medium without In_2_(SO_4_)_3_ have a circularity value of 0.28 ± 0.08 (*n* = 140 cells), which is ~43% smaller than cells treated with 3.2 mM of indium, which has a circularity value of 0.49 ± 0.11 (*n* = 286 cells). The data spread for these values, which corresponds to one standard deviation, indicates that cells become more circular in shape upon indium exposure.

(1) $$\mathrm{circularity}\;\mathrm{index}=4\pi \frac{\mathrm{cell}\;\mathrm{projected}\;\mathrm{area}}{{\left(\mathrm{cell}\;\mathrm{primeter}\;\mathrm{length}\right)}^{2}}$$

One possible explanation for the change in cell morphology is that In_2_(SO_4_)_3_ could induce a change in cellular and/or subcellular organellar stiffness, as described for compounds such as Trichostatin A (TSA) and heavy metal, in the form of lead nitrate, in studies by Fischer et al. [37] and Zhu et al. [38], respectively. Fischer et al. showed that the chromatin de-condensing reagent TSA can decrease the stiffness of the cytoskeleton and nuclei in MCF-7 breast cancer cells. In contrast, Zhu et al. reported that the stiffness of Madin Darby canine kidney cells (MDCK NBL-2) increased by ~65% after exposure to 1 mM lead nitrate [38]. Exposure to In_2_(SO_4_)_3_ may produce a similar cellular stiffening as lead. 

Since heavy metal ions are known to interact with chromatin in the nucleus [39], and the shape of nuclei is affected by mutated lamin proteins [40], it is also important in adherent cells. The aspect ratio between the length and width of nuclei was characterized, and the results are summarized in Table 1. Data show the averaged aspect ratios of nuclei, regardless of treatments, are statistically indistinguishable and in the range of 1.50 and 1.57, even though the overall cellular shape becomes more circular with In_2_(SO_4_)_3_ treatment (see Figure 2). The results suggest that the influence of In_2_(SO_4_)_3_ on the morphology of the cell nuclei is not coupled to the overall shape of the cells. 

### 3.2. Cell Viability 

**Figure 3:**
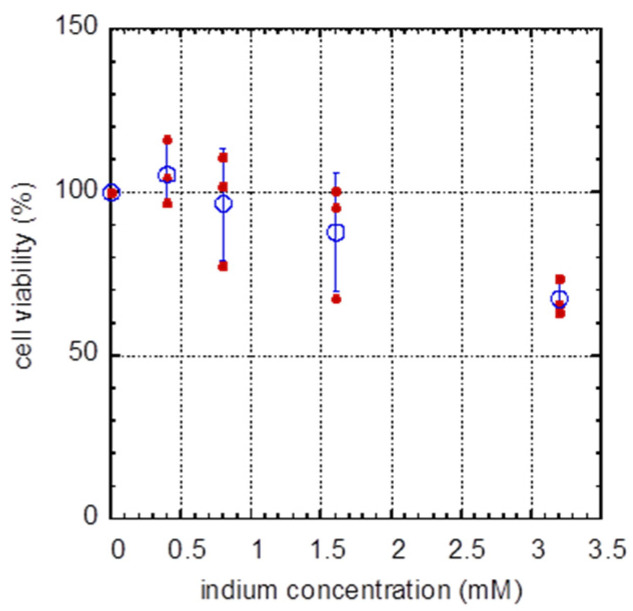
Dose-dependent cell viability after In_2_(SO_4_)_3_ treatments. Results show that cell viability reduces with the increased concentration of indium in the media. Experiments were performed in triplicate. Solid circles indicate results from individual experiments, while the open circles represent their average values. Data spread corresponds to one standard deviation. Results show that cell viability decreases with indium concentration.

As shown by the fluorescence confocal micrographs presented in Figure 2, there appeared to be fewer adherent cells in culture dishes after incubating in media containing 3.2 mM of indium. To quantify this apparent In_2_(SO_4_)_3_ -induced degradation on the health of cells, dose-dependent cell viability experiments were conducted using alamarBlue assays (Figure 3). Cell viability data are expressed as a function of indium concentration rather than In_2_(SO_4_)_3_ to conform with the practice in other publications [22]. Experiments were carried out in triplicate for each indium concentration, and the viability was represented by the solid circles; the average viability for each indium concentration is represented by the open circle, with the corresponding standard deviation also included. While the change in viability is undetectable or statistically insignificant when the dosage is lower than 1.6 mM, at an indium concentration of 3.2 mM, the average dermal cell viability is ~68% which is significantly lower than the viability of samples without In_2_(SO_4_)_3_ treatment (0 mM), p = 0.0006. Similar viability of GM5565 cells (~65%) was observed by Eskandari et al. [30] when cells were incubated in media containing 3.2 mM InCl_3_. These results suggest that there is no significant difference in the impact of the counter ions (Cl^−^ vs. SO_4_^2−^) on cell viability. Compared to the viability of other cell types upon treatment with the same concentration of indium, the viability of immortalized human oral keratinocytes (IHOK and HSC-2) is ~77% and ~88% [22], respectively. The difference in viability between the GM5565 cells and the HSC-2 cell line is likely a reflection of the type of cell, as well as the untransformed fibroblasts compared to transformed cancer cells, respectively. 

While the cell viability decreases with In_2_(SO_4_)_3_ supplement, the exact role of indium in the degradation of the cell’s health is unclear. Some indium ions may have interacted with other chemical components in the media, such as negatively charged protein and lipid molecules. These indium complexes may affect cells differently from free indium ions. It is also possible that indium reacts with phosphate and form precipitates or aggregates, which may also reduce cell viability. Future work will be needed to investigate the detailed viability degradation mechanism induced by In_2_(SO_4_)_3_.

### 3.3. Indium Sulfate-Induced Cellular ROS Production

Several studies [19,22,41,42] have shown that the accumulation of cellular ROS leads to oxidative stress, DNA damage, and cardiovascular disease. Given that one of the sources of ROS in human fibroblasts is the mitochondrial respiratory chain, we interrogated the morphology of the mitochondria in live GM5565 cells to characterize the effects of In_2_(SO_4_)_3_. In order to probe the mitochondrial reticulum in cells, we analyzed MitoTracker Red using fluorescence confocal microscopy. 

**Figure 4:**
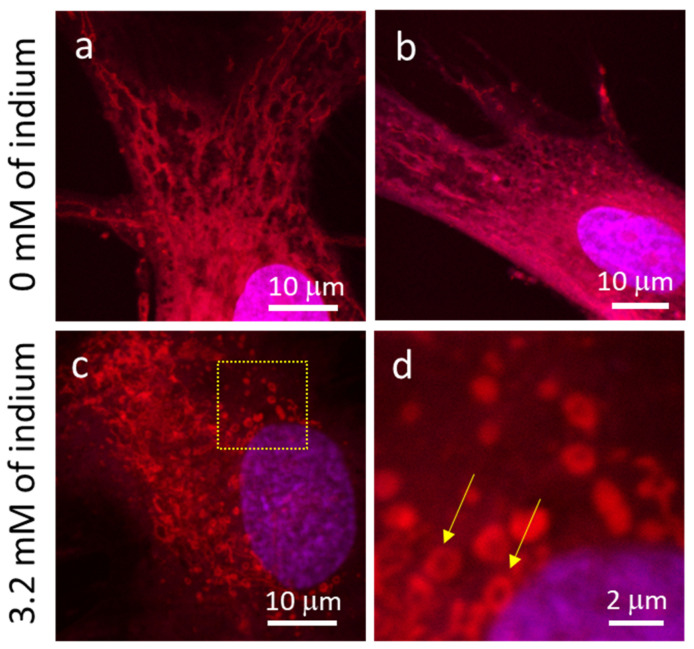
Typical fluorescence confocal micrographs of cells incubated in media containing (**a**,**b**) 0 mM and (**c,d**) 3.2 mM of indium. Mitochondria in cells are labelled with MitoTracker Red, and the DNA molecules with blue DAPI. Micrographs show that the mitochondria form extended tubular networks in cells incubated in indium-free media (**a**,**b**). The mitochondrial morphological change to puncta when cells were incubated in a medium containing 3.2 mM indium (**c**,**d**). Some puncta of mitochondria are highlighted with arrows in the figure.

Typical micrographs of adherent cells in indium-free and In_2_(SO_4_)_3_-containing media are displayed in Figure 4a–d, respectively, revealing the expected network of the mitochondrial reticulum in cells prepared from indium-free media (Figure 4a,b). In contrast, mitochondrial staining is predominantly punctate after cells were exposed to In_2_(SO_4_)_3_. These changes from tubular networks to punctate staining in mitochondrial morphology were also observed when GM5565 cells were exposed to a known ROS-inducing agent, Antimycin A [28], suggesting that In_2_(SO_4_)_3_ may induce intracellular ROS production.

**Figure 5:**
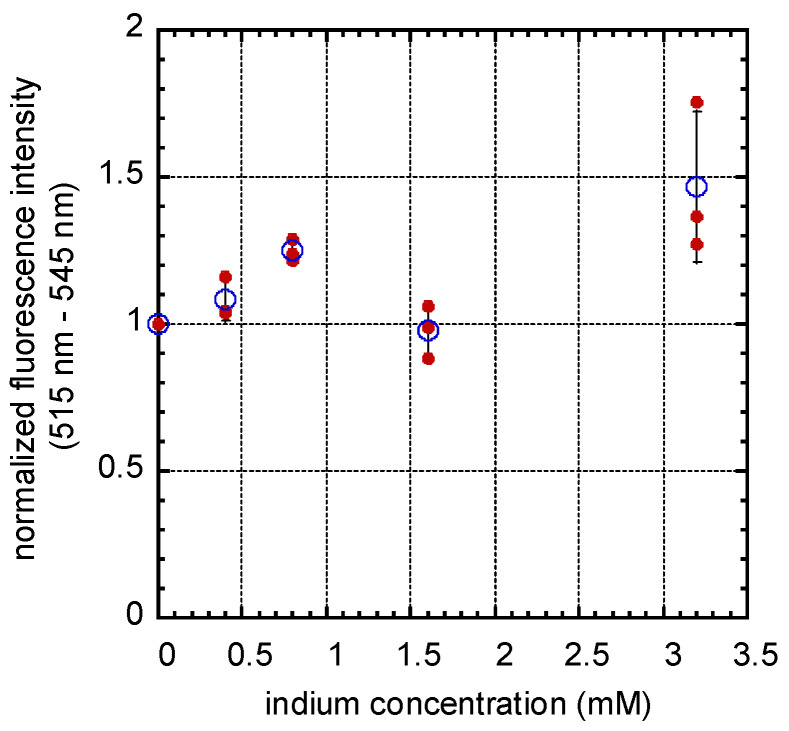
Indium sulfate-induced dose-dependent response on DCFDA fluorescence intensity. Cells were incubated in indium sulfate-containing media for 24 h and ROS probe DCFDA for 30 min. Samples were characterized using an excitation wavelength of 485 nm, and the fluorescence intensity with wavelengths in the range of 515 nm–545 nm was measured. Experiments were performed in triplicate. Solid circles indicate results from individual experiments, while the open circles represent their average values. Data spread corresponds to one standard deviation. Results suggested that indium sulfate induces ROS production in fibroblasts.

To ascertain whether In_2_(SO_4_)_3_ induced ROS production, cells incubated with media containing 0 mM, 0.4 mM, 0.8 mM, 1.6 mM, and 3.2 mM of indium were assayed with the ROS probe 2′,7′-dichlorofluorescein diacetate (DCFDA) and the dose-dependent effect of In_2_(SO_4_)_3_ on fluorescence intensity of DCFDA in cells is displayed in Figure 5. The fluorescence intensity results were normalized to the signal from cells incubated in indium-free medium and to the cell viability values to account for a reduction in cell numbers in In_2_(SO_4_)_3_ treated specimens. The solid circles represent the data from each individual assay, which was performed in triplicate. The open circles and error bars indicate the average intensity value for each specific indium concentration and the data spread of one standard deviation, respectively. While the data suggest that increasing levels of ROS are associated with increasing indium concentrations, the lack of increased ROS at 1.6 mM indium is puzzling, and we do not currently have a cogent explanation for this outlier. In general terms, these results show that the normalized DCFDA intensity increases with the indium concentration and support the notion that In_2_(SO_4_)_3_ induces ROS production in GM5565 cells. 

### 3.4. Indium Sulfate Induced-Cell Adherent Behaviors on Engineered Surfaces

The results presented in Figure 4 and Figure 5 strongly suggest that cells contain higher ROS levels when exposed to In_2_(SO_4_)_3_, which may lead to a change in cell adhesion characteristics. For example, Moussa et al. [28] have shown that the morphology and population of cells aligned to the tungsten/silicon oxide parallel line surface structures changed significantly when cells were treated with the mitochondrial respiratory inhibitor, Antimycin A, which is a biotoxin known to induce ROS production in cells. Hence, the GM5565 cell alignment on engineered surfaces could be expected to be impacted by the presence of In_2_(SO_4_)_3_ in the media. To test this hypothesis, we characterized the In_2_(SO_4_)_3_ dose-dependent cell adhesion behaviors on engineered surfaces of parallel line/trench structures. The sidewall and trench bottom surfaces are coated with Ta, while the line surface consists of silicon oxide (see Figure 1).

**Figure 6:**
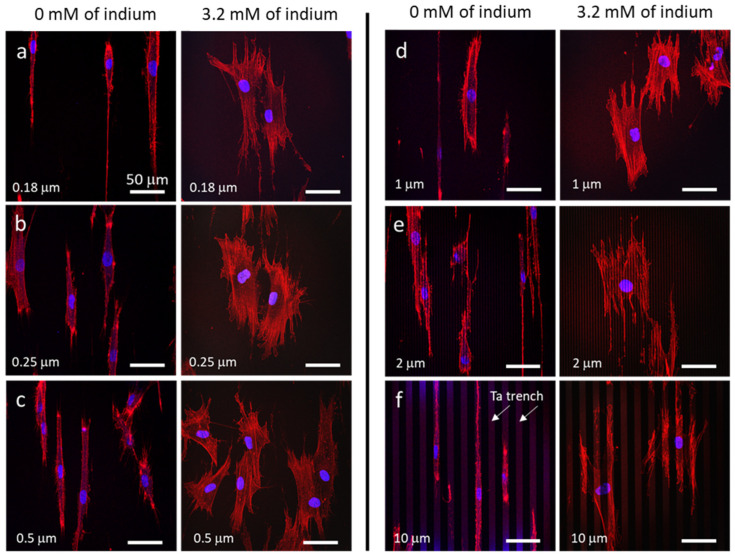
Typical confocal fluorescence micrographs displaying cell alignment characteristic on parallel line structures with equal trench/line widths of (**a**) 0.18 μm, (**b**) 0.25 μm, (**c**) 0.5 μm, (**d**) 1 μm, (**e**) 2 μm, and (**f**) 10 μm. The left panels show cells incubated in indium-free media, while the right panels display cells treated with indium sulfate (3.2 mM of indium). Microfilaments were labelled with red phalloidin, while DNA molecules were stained with DAPI. The trenches and lines are oriented vertically in these micrographs. The scale bars correspond to 50 μm.

Representative confocal micrographs of adherent cells incubated in media with (3.2 mM indium) and without In_2_(SO_4_)_3_ on parallel line structures are displayed in Figure 6a–f. The figure shows adherent cells on structures with line/trench widths of 0.18 μm, 0.25 μm, 0.5 μm, 1 μm, 2 μm, and 10 μm, respectively, with the trenches and lines oriented vertically. Since tantalum reflects light more than silicon oxide, the trench structures appear brighter than the lines. Micrographs show adherent cells elongated and aligned to the line axes when incubated in indium-free media (left panel). These cells maintained an elliptical or tear-drop shape with long actin microfilaments (red) extended to the opposite ends of the cells.

In contrast, bundles of short microfilaments extended from compact-shaped cells when treated with In_2_(SO_4_)_3_ (right panel). While the micrographs show a significant change in the shape of cells upon indium treatment, the short actin microfilaments continue to extend in the direction parallel to the line axes. Careful inspections of the micrographs in Figure 6 show that, regardless of the medium composition, the actin microfilaments preferentially extend along trenches with widths of 1 μm, 2 μm, and 10 μm, where the sidewall and bottom surfaces are coated with a thin layer of Ta. This suggests that cellular actin filaments maintain mobility and preferential alignment behavior even when exposed to media containing In_2_(SO_4_)_3_ with 3.2 mM of indium. The lack of elongation for cells exposed to In_2_(SO_4_)_3_ could be due to metal ion-induced cell stiffening [38,39], given that as the stiffness of cellular materials increases, a larger amount of mechanical force is required to deform or elongate the cell.

### 3.5. Interplay between Indium Sulfate and Surface Topographic Patterns in Driving Cell Morphology

**Figure 7:**
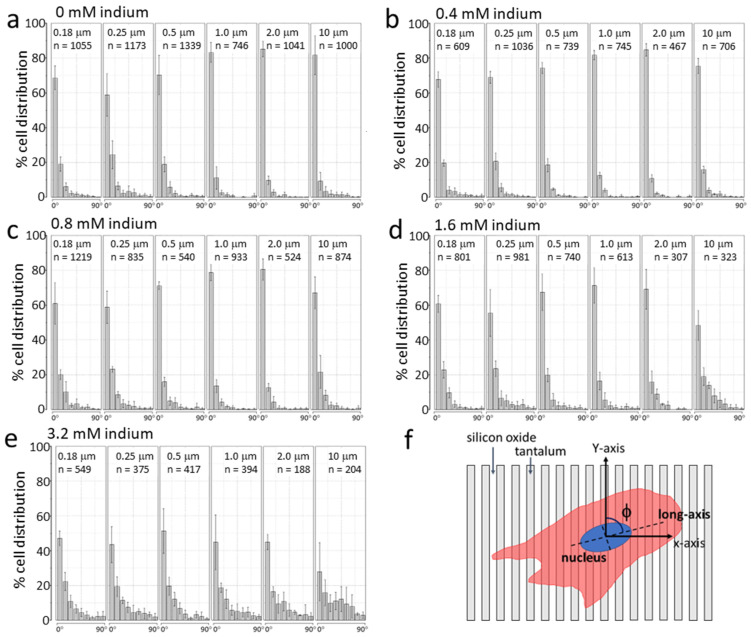
Cell alignment behaviors on parallel line structures with equal line and trench widths of 0.18 μm, 0.25 μm, 0.5 μm, 1 μm, 2 μm, and 10 μm. Panel (**a**) shows the distribution of cells aligned on the structures when cultured in an indium-free media (0 mM of indium). Population data from cells prepared with media containing 0.4 mM, 0.8 mM, 1.6 mM, and 3.2 mM of indium are shown in panels (**b**–**e**), respectively. Each plot shows data of cell orientation measured from at least three blocks of areas, and the data spread corresponds to one standard deviation. Results indicate fewer cells aligned to the line axes when treated with indium sulfate. A schematic drawing of a cell on the parallel line structure and the measured angular displacement (ϕ) is shown in (**f**).

Our results thus far demonstrate that cell viability decreases, and cellular and mitochondrial morphologies change when GM5565 cells are exposed to In_2_(SO_4_)_3_. However, some cellular functions remain seemingly unperturbed. For example, the actin filaments from cells treated with In_2_(SO_4_)_3_ continue to extend preferentially along the engineered structure line axes, as shown in Figure 6. It is unclear if this micro-scale cytoskeletal mobility is sufficient to orient the entire cell into the line axes. Furthermore, there is a lack of information regarding the complex interplay between the concentration of In_2_(SO_4_)_3_ in the culture medium and surface topographic patterns in determining subsequent cell morphologies. To understand this multi-variable-induced impact on cells, we measured the orientation of adherent cells on parallel line structures consisting of line/trench widths of 0.18 μm, 0.25 μm, 0.5 μm, 1 μm, 2 μm, and 10 μm after incubating them in media containing 0 mM, 0.4 mM, 0.8 mM, 1.6 mM, and 3.2 mM indium. Cell alignment on the parallel line structures was quantified by measuring the angular displacement (ϕ) between the long axis of the cell nucleus and line axes (as defined in Figure 7). 

Indium dose-dependent cell alignment behaviors on parallel line structures with specific line/trench widths are shown in Figure 7. The orientation of cells on at least three blocks of the area was characterized by each specific patterned structure and indium concentration. The number of cells characterized (n) for each experimental condition is indicated in the resultant plot, and each bin represents the population of cells within a 10° increment of angular displacement. For example, the distribution of cells aligned from 21° to 30° and −21° to −30° is represented by the third bin. In total, this approach characterized the orientation of more than 20,000 cells. Our analysis shows that cell alignment characteristics depend on the dimension of the line/trench widths and the indium concentration in the cell culture medium. When adherent cells on parallel line structures with widths of 0.18 μm, 0.25 μm, and 0.5 μm are incubated with indium-free medium, as shown in Figure 7a, ~59% to ~70% of the averaged cell population aligns to the line axes, which is defined as the distribution of cells that are oriented to within ±10° of the lines. The population of aligned cells increases to ~80% to 85% on structures with line/trench widths of 1 μm, 2 μm, and 10 μm. This demonstrates a surface topography-induced cell alignment effect when prepared with indium-free media. 

When cells are treated with In_2_(SO_4_)_3_, the population of cells aligned to the line axes decreases with an increased dosage of indium (Figure 7b–e). Interestingly, this In_2_(SO_4_)_3_-induced cell alignment behavior is more prominent on structures with line/trench widths of 1 μm, 2 μm, and 10 μm than those with widths of 0.18 μm, 0.25 μm, and 0.5 μm. For example, the average population of adherent cells on the structures with line/widths of 0.18 μm aligned to the line axes decreased from ~69% to ~47% on exposure to 0 mM and 3.2 mM indium, respectively. When cells are exposed to the same indium concentration on structures with line/trench widths of 10 μm, the proportion of aligned cells decreases dramatically from ~82% to ~28%. This demonstrates that the pattern-dependent cell alignment performance can be affected by the concentration of indium in the media. Adherent cells on structures with line/trench widths of 1 μm, 2 μm, and 10 μm appear to be impacted by the In_2_(SO_4_)_3_ more significantly than those on structures with line widths smaller than 0.5 μm. The decrease in the GM5565 cells’ ability to align on the parallel line structures may be due to the increase in ROS production in cells when exposed to In_2_(SO_4_)_3_. Moussa et al. [28] reported a surmised ROS-dependent cell alignment effect in GM5565 cells after they were incubated in media containing Antimycin A. Those results showed that the population of cells aligned to the parallel line structures, consisting of dissimilar materials (tungsten and silicon oxide), significantly decreased when cells were treated with Antimycin A. 

**Figure 8:**
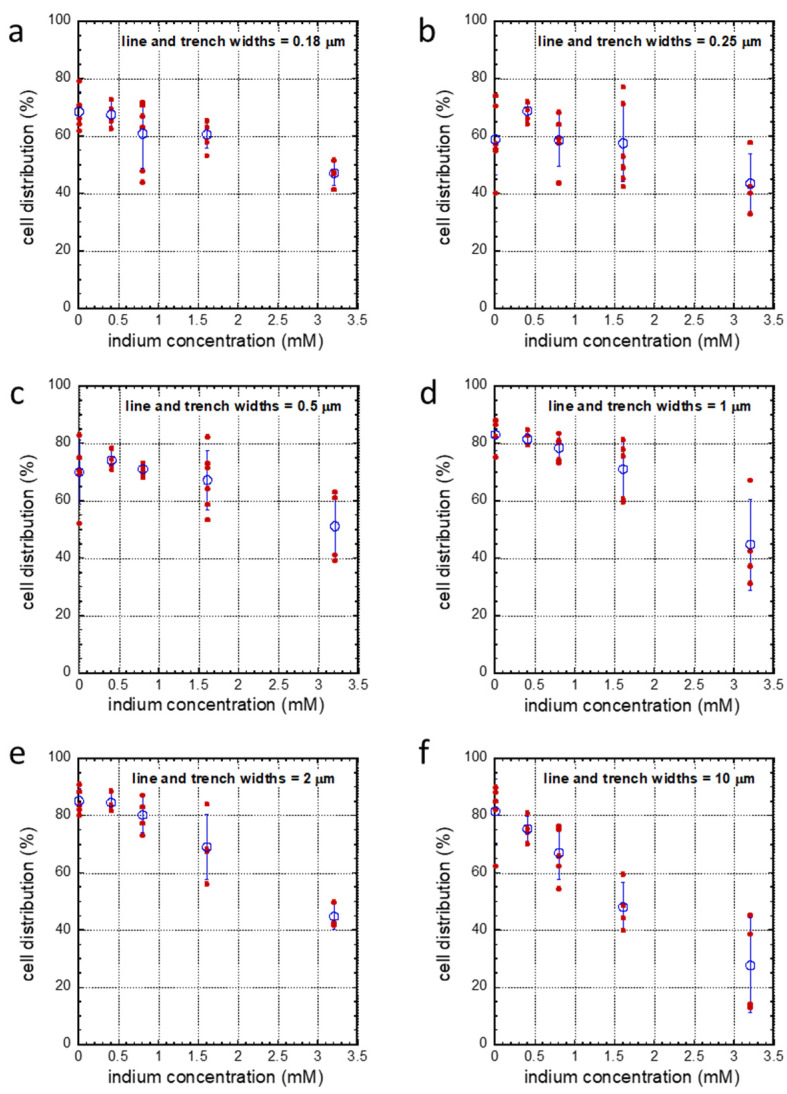
Decoupling between the effects of parallel line patterns and indium concentration in the cell culture media on cell alignment to the line axes. Equal trench/line widths (**a**) 0.18 μm, (**b**) 0.25 μm, (**c**) 0.5 μm, (**d**) 1 μm, (**e**) 2 μm, and (**f**) 10 μm. Results show the degradation of the cell alignment performances with the dosage of indium. The amount of degradation is more pronounced for adherent cells on structures with wider line/trench widths. Solid circles indicate results from individual experiments, while the open circles represent their average values. Data spread corresponds to one standard deviation.

**Figure 9:**
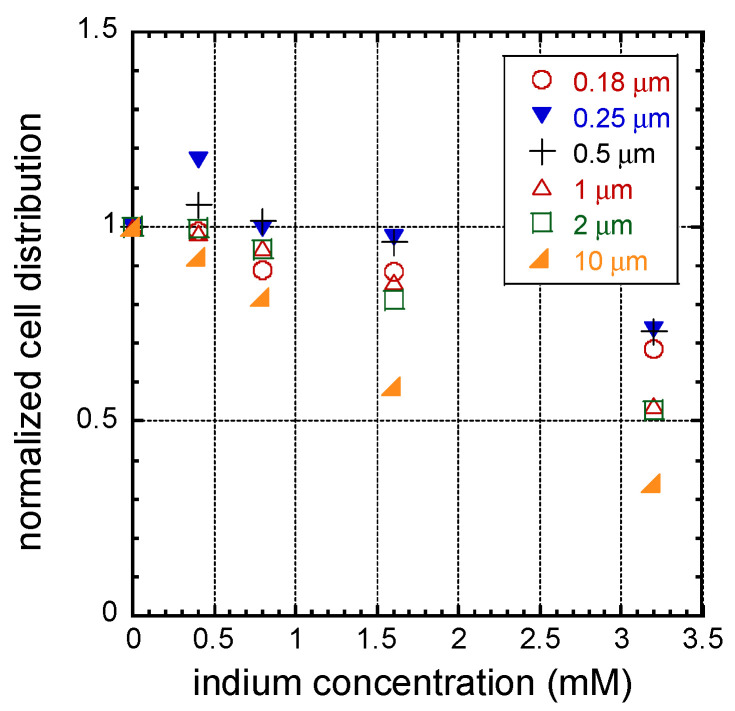
Normalized cell distribution of cells aligned on parallel line structures when prepared in media containing different concentrations of indium in the media.

To further decouple the surface topographic effect from the In_2_(SO_4_)_3_ dosage effect on cell alignment, the population of cells aligned within ±10° of the line axes from each specific pattern was plotted as a function of indium dosages in Figure 8a–f. Solid symbols indicate the population of cells aligned to the pattern from each block of sampled area, while open circles represent the averaged result from each group of samples, and the error bars indicate one standard deviation. The data show a general trend of decreasing cell alignment with increasing indium concentration. The impact of In_2_(SO_4_)_3_-induced change in cell alignments appears small and insignificant when cells are incubated on structures with trench/line widths smaller than 0.5 μm (Figure 8a–c, respectively). In contrast, there is a significant decrease in the proportion of cells oriented parallel to the pattern axes on structures with trench/line widths of 2 μm and 10 μm (see Figure 8e,f). This pattern-dependent indium sulfate-induced cell alignment degradation is further highlighted in Figure 9, in which the averaged proportions of aligned cells were normalized to the alignment result from cells incubated in indium-free media. This plot clearly shows a ~31% decrease in the alignment of cells on structures with line/trench widths of 0.18 μm, while there is a ~72% decrease in the cell population that aligns on structures with line widths of 10 μm. This indicates that the impact of In_2_(SO_4_)_3_ on cells increases with line/trench width, at least up to 10 μm; future studies are needed to determine if this pattern dependence continues for structures with larger line/trench widths.

### 3.6. Influence on Nuclear Morphology of Indium Sulfate and Surface Topographic Patterns

The results from Section 3.5 show the interplay between In_2_(SO_4_)_3_ and engineered surface patterns and their effect(s) on the overall adherent cell morphology. However, as described in Section 3.1, the influence of In_2_(SO_4_)_3_ on overall cell morphology (circularity index) does not appear to extend to the nucleus. Since the nucleus is the largest organelle in animal cells and heavy metal ions can react with chromatin and nuclear membranes [39,40], it is important to consider whether these external cues might impact the morphology of the nucleus. While the aspect ratio of nuclei in adherent cells on flat petri dish surfaces does not show a statistically significant change with indium treatments (see Table 1), it was important to determine whether this behavior would hold for cells adhered to engineered surfaces.

Therefore, we determined the aspect ratio of nuclei in adherent cells on parallel line engineered surfaces, with and without indium treatments (Table 2). The table shows the averaged aspect ratio of nuclei in cells incubated in the indium-free medium is in the range of 1.98–2.24, indicating that nuclei in these cells are proportionally longer along the major axis than those in cells on the flat petri dish surfaces (aspect ratio of ~1.50). A possible explanation for this surface-engineered pattern-induced change in nuclear morphology could be that, as the cell aligns itself to the patterned metal (see Figure 6), the cytoskeleton re-orients itself and the nucleus into the same alignment direction. When cells were incubated in a medium containing In_2_(SO_4_)_3_, Table 2 shows that the aspect ratio of nuclei in cells adhered on the engineered structures was in the range of 1.59–1.88, which is smaller than those measured from cells incubated with indium-free media. This reflects a decreased elongation of the overall cellular structure and subsequent deformation of the nucleus in cells exposed to indium. Our results suggest that the impact of In_2_(SO_4_)_3_ on nuclear morphology is influenced by the nano-/micro-structures on the substratum. For smooth Petri dishes, there is no discernible change in nuclear morphology in the face of an overall change in cell shape, while changes in the shape of nuclei are detectable for cells adhered to the parallel line structures. 

The results presented in this work demonstrate that the degree of impact of In_2_(SO_4_)_3_ on human fibroblasts depends on the topography of the substratum to which they adhere. Since cells in multi-cellular organisms interact with the ECM, which consists of a wide variety of topographic features that depend on cell, tissue and organ type, experimental data collected on flat surfaces should not be considered representative. Likewise, the vast majority of studies regarding the response of cells to toxic contaminants have been carried out on adherent cells on flat substrate, and the results we present here suggest the need for extending studies of environmentally-important or human health-related contaminants to textured surfaces, characterizing cell behaviors on a wide range of such surfaces is needed to develop a better understanding of the impacts of external contaminants on cell behavior and the potential interactions with implants. 

## 4. Conclusions

The influence of In_2_(SO_4_)_3_ on human dermal fibroblast viability, morphology, and ROS production was characterized in this study. Our results show cell viability decreases and cellular ROS production increases with increasing concentrations of In_2_(SO_4_)_3_ in the media. Although the actin microfilaments continue exhibiting preferential adhesion to the Ta-coated trenches, even after cells are exposed to In_2_(SO_4_)_3_, the cytoskeleton within cells treated with indium is not efficient in orienting the cells to the line axes. We describe a decrease in the proportion of cells aligned to the line axes upon In_2_(SO_4_)_3_ exposure. For cells treated with the same indium dosage, the degradation in the ability of cells to align is larger on structures with line/trench widths in the range of 1 μm and 10 μm than those with line widths smaller than 0.5 μm. Our analyses show that the impact of In_2_(SO_4_)_3_ on cells is sensitive to the surface topography of the substratum and suggest that experiments designed to better understand how cells are impacted by external chemical cues would benefit from the use of textured surfaces as substrata in such studies.

## Figures and Tables

**Table 1:** The average aspect ratio of nuclei in adherent cells when incubated in a medium without In_2_(SO_4_)_3_ and a medium containing 3.2 mM of indium, respectively. Data spread corresponds to one standard deviation.

Indium Concentration (mM)	Aspect Ratio	Standard Deviation	*n*
0	1.50	0.28	20
3.2	1.57	0.36	33

**Table 2:** Results of nucleus aspect ratio in cells adhered on engineered structures without and with In_2_(SO_4_)_3_ treatments. Data spread corresponds to one standard deviation (S.D.).

Indium Concentration	Trench/Line Width	Aspect Ratio	S.D.	*n*
(mM)	(μm)			
0	0.18	2.06	0.45	61
0	0.25	1.98	0.46	74
0	0.5	2.05	0.35	63
0	1	2.24	0.69	54
0	2	2.11	0.51	91
0	10	2.14	0.51	68
3.2	0.18	1.63	0.39	54
3.2	0.25	1.71	0.43	44
3.2	0.5	1.74	0.35	60
3.2	1	1.88	0.45	39
3.2	2	1.72	0.38	27
3.2	10	1.59	0.3	26

## Data Availability

The data presented in this study are available on request from the corresponding author.
